# The outcomes and usefulness of Intraoperative Optical Coherence Tomography in vitreoretinal surgery and its impact on surgical decision making

**DOI:** 10.22336/rjo.2022.12

**Published:** 2022

**Authors:** Rayyan Zakir, Kashif Iqbal, Muhammad Hassaan Ali, Umair Tariq Mirza, Khalid Mahmood, Sidrah Riaz, Nauman Hashmani

**Affiliations:** * Layton Rahmatullah Benevolent Trust, Lahore, Pakistan; ** Allama Iqbal Medical College, Jinnah Hospital, Lahore, Pakistan; *** Mohiuddin Islamic Medical College Mirpur, Azad Jammu & Kashmir; **** Avicenna Medical College and Hospital, Lahore, Pakistan; ***** Akhtar Saeed Medical and Dental College, Lahore, Pakistan; ****** Hashmanis Group of Hospitals, Karachi, Pakistan

**Keywords:** intraoperative optical coherence tomography, vitreoretinal surgery, Leica Enfocus, retinal detachment, macular hole

## Abstract

**Objective.** To evaluate the utility of intraoperative OCT and its influence on the surgeon’s decision during vitreoretinal surgery.

**Methods.** This was a pilot, prospective case series conducted at a tertiary care ophthalmology department in Lahore, Pakistan. Sixteen patients undergoing vitreoretinal surgeries were included using the *Leica Enfocus* microscope integrated intraoperative optical coherence tomography (IOCT). We also investigated the changes in surgical decision making based on the findings revealed by IOCT using a questionnaire.

**Results.** 16 patients with a mean age of 40.6 ± 19.0 (range: 11-66) years, were included in the study; one case of acute postoperative endophthalmitis could not be imaged. The surgeon had to modify surgical decisions in four (26.7%) cases. IOCT clearly delineated various tissue planes for efficient and safe surgical dissection in pathologies such as posterior vitreous detachment, vitreomacular traction and epiretinal membranes. Furthermore, it also helped identifying perfluorocarbon-retina interface.

**Conclusions.** The intraoperative OCT modality is a feasible and useful intraoperative imaging technique for various kinds of vitreoretinal disorders. The decision making of the surgeon was modified in a quarter of the cases after the use of this newer modality.

## Introduction

Optical coherence tomography (OCT) is an imaging modality that performs cross-sectional imaging of ocular tissues by measuring the magnitude and echo time delay of backscattered light from target ocular structures [**1**]. This modality has a vital role in the management of retinal diseases and therefore, it is prudent to consider its utilization and integration in the operating room through microscope integrated intra-operative OCT (IOCT) [**2**]. 

Previous studies have reported the utility of IOCT in various anterior and posterior segment surgeries. These include the landmark PIONEER and DISCOVER studies that used microscope mounted and microscope integrated OCT modules for IOCT [**3**,**4**]. Most of the studies have been reported by resource rich institutions with reasonable patient load and access to newer technologies [**5**]. Additionally, earlier studies utilized an external OCT system that required the surgeon to pause the surgical procedure to perform imaging and then take subsequent surgical decisions [**3**]. 

We report our experience with a microscope integrated IOCT system for real time imaging and tissue-instrument interaction on OCT during various vitreoretinal procedures. We aimed at highlighting the role and feasibility of the IOCT to delineate retinal structures and its ability to influence the surgeon’s decision-making process. 

## Method

This was a prospective, consecutive case series performed by a single surgeon (KI) in a single center. We evaluated the utility and feasibility of IOCT (Bioptigen/ Leica Microsystems, Wetzlar, Germany) in vitreoretinal surgery. The study was conducted after taking approval from the Institutional Review Board of *Layton Rahmatullah Benevolent Trust*, Lahore, Pakistan and adhered to the tenets of the Declaration of Helsinki. All patients were recruited after signing a written informed consent. For those who were under 18 years old, an informed consent was taken from the parents. 

The inclusion criteria of our study referred to patients with a condition requiring vitreoretinal surgery, willing to undergo IOCT and either themselves or a parent were able to provide a written informed consent. All types of vitreoretinal surgeries were included. 

All the surgical cases were operated with the *Leica Enfocus* operating microscope integrated IOCT system. This is one of the few commercially available systems that employs intraoperative OCT and gives real time per-operative imaging of the retina without having to stop the surgery.

All the diagnosis and advised surgical procedures were recorded in a pre-designed questionnaire. The operating surgeon was asked to record specific findings of the IOCT after each surgery encompassing the following three areas: identifying important vitreoretinal structures during the procedure, the impact in altering the surgeon’s decision and confirmation of the surgeon’s decision preoperatively. Questions were also asked if the surgeon had to modify the surgery considering the findings revealed by the IOCT imaging. 

The statistical analysis was performed using Statistical Package for Social Sciences (SPSS version 25.0, IBM Statistics, Chicago, IL, USA). Numerical variables were calculated as mean ± standard deviation (SD), whereas descriptive variables were calculated in frequencies and percentages. 

## Results

A total of 16 patients, with a mean age of 40.6 ± 19.0 (range: 11-66) years, and a female to male ratio of 1:1.3, were included in the study. The most common vitreoretinal condition treated was retinal detachment (10, 62.5%). The details are provided in **Table 1**.

**Table 1:** The utility of Intraoperative OCT in each surgery

No.	Age (years)	Diagnosis and Relevant Retinal Findings	Vitreoretinal Surgery	Specific Indication of Using Intraoperative OCT		Utility of Intraoperative OCT	
					Identification of Important Retinal Structures	Surgeon’s Decision Making	Confirmation of Surgeon’s Surgical Decision
1	60	Retinal Detachment	Pars plana vitrectomy (PPV) with silicon oil tamponade	To rule out any macular pathology such as macular edema or AMD	Yes	No	Yes
2	16	Re-Retinal Detachment	Redo PPV with silicon band	To rule out sub-retinal oil preoperatively and confirmation of retinal attachment at the end of surgery	Yes	No	Yes
3	45	Rhegmatogenous Retinal Detachment	PPV with Silicon Oil	To confirm presence of detachment and to rule out any sub-retinal oil after the procedure	Yes	No	Yes
4	40	Myopic Retinal Detachment with Proliferative vitreoretinopathy (PVR) and multiple retinal breaks.	PPV with Silicon Oil	To confirm the presence of epiretinal membrane (ERM) and completion of posterior vitreous detachment as vitreous was very adherent	Yes	Yes	Yes
5	66	Inferior Bullous Retinal Detachment with extensive macular scarring	Inferior Band + PPV with Silicon Oil	To confirm the cause of macular scarring, which turned out to be active choroidal neovascularization and ensure complete removal of perfluorocarbon	Yes	Yes	Yes
6	61	Diabetic vitreous hemorrhage with Tractional Retinal Detachment (TRD)	PPV with Silicon Oil	To ascertain the location of TRD and residual ERMs	Yes	Yes	Yes
7	30	Emulsified Silicon Oil causing Glaucoma	Removal of Silicon Oil	Removal of silicon oil revealed macular hole, which was confirmed using intraoperative OCT	Yes	No	Yes
8	28	Traumatic Retinal Detachment with ERM	PPV with Silicon oil	To confirm retina attachment and any remnant ERM after ERM Peeling	Yes	No	Yes
9	24	Total Retinal Detachment with PVR	Band + PPV + Silicon Oil	To determine the presence of any ERM and confirmation of complete removal of heavy liquid and retinal attachment at the end	Yes	No	Yes
10	57	Diabetic Vitreous Hemorrhage	PPV	To detect macular edema and other associated pathologies like diabetic TRD and ERM	Yes	Yes	Yes
11	60	Post-operative Endophthalmitis	Core Vitrectomy with Silicon Oil	To determine any retinal traction since the view was hazy	No	No	No
12	15	Retinal Detachment with very adherent PVD	PPV + Silicon Oil	To assess completion of PVD & any residual traction on the macula	Yes	No	Yes
13	27	Diabetic TRD with extensive ERMs	PPV + Silicon Oil	To confirm any remnant retinal tractions and ensure complete removal of ERMs	Yes	No	Yes
14	48	Epiretinal Membrane causing refractory diabetic macular edema	PPV + C3F8 gas	To confirm complete removal of ERM	Yes	No	Yes
15	11	Traumatic Macular Hole	PPV + Internal Limiting Membrane (ILM) Peel with C3F8 gas	To confirm completion of PVD in young highly adherent vitreous and determination of macular hole morphology after ILM Peel	Yes	Yes	Yes
16	61	Idiopathic Macular Hole	PPV + ILM Peel + SF6 gas	To confirm completion of PVD and determination of hole morphology after ILM Peel	Yes	No	Yes

Successful image acquisition was obtained in 15 cases (93.8%). In these cases, 100% of the images identified important anatomical structures during surgery. A case with acute postoperative endophthalmitis after cataract surgery yielded poor images that could not be analyzed. 

**IOCT caused a direct change in decision**

**Fig. 1:**
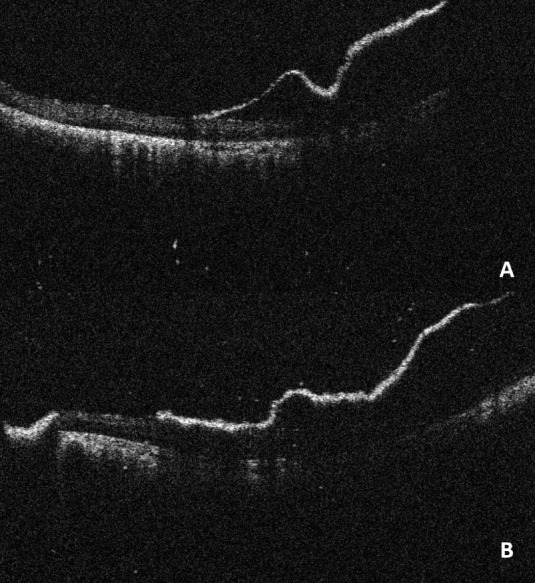
**(A)** Vitreous is still attached temporal to the disc; **(B)** Vitreous was then detached before closure

The IOCT images helped modify the surgeon’s decision making in four cases (26.7%). In two cases of vitrectomy in young patients, the IOCT helped visualize highly adherent vitreous at the posterior pole temporal to the disc, which was not apparent on the surgical microscope. An example is shown in **Fig. 1**. The vitreous was then detached, thus completing the PVD before proceeding to the next step. 

**Fig. 2:**
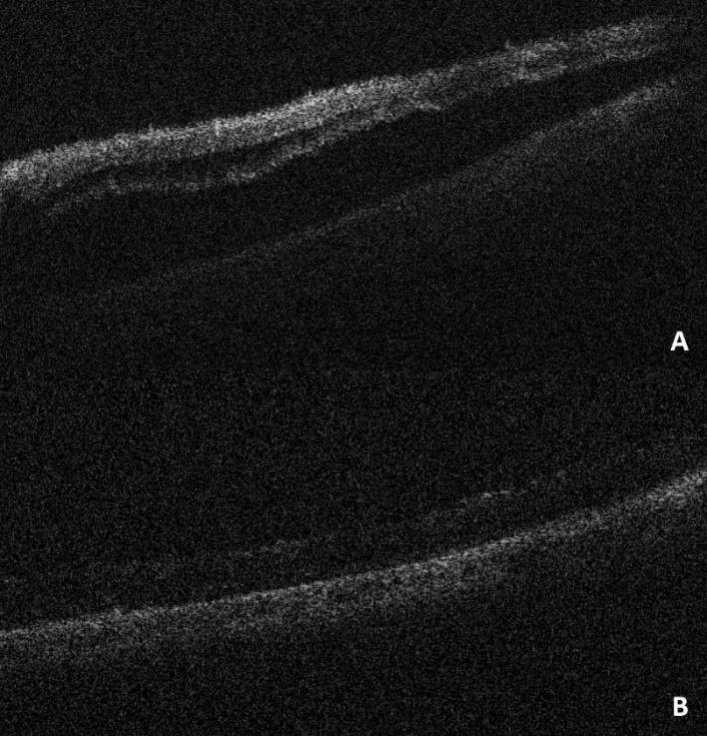
**(A)** Evidence of subretinal fluid post-surgery without the help of the intraoperative OCT. **(B)** Re-attached retina post drainage of the subretinal fluid

In other two cases, IOCT helped identify mild subretinal fluid in cases of retinal detachment with an apparently reattached retina. In these cases, the subretinal fluid was drained and a reattached retina was confirmed. One of these is shown in **Fig. 2**. 

**IOCT aided in the decision-making process**

In two cases of proliferative vitreoretinopathy (PVR), the modality helped image the extent and location of epiretinal membranes (ERMs). Additionally, the IOCT helped view subretinal fibrosis and the presence of any subretinal oil intraoperatively. 

In cases of retinal detachment, IOCT was helpful in the confirmation of reattachment, and therefore, the injection of oil could be made with confidence. Additionally, it helped confirm the complete removal of perfluorocarbon heavy liquid.

**Fig. 3:**
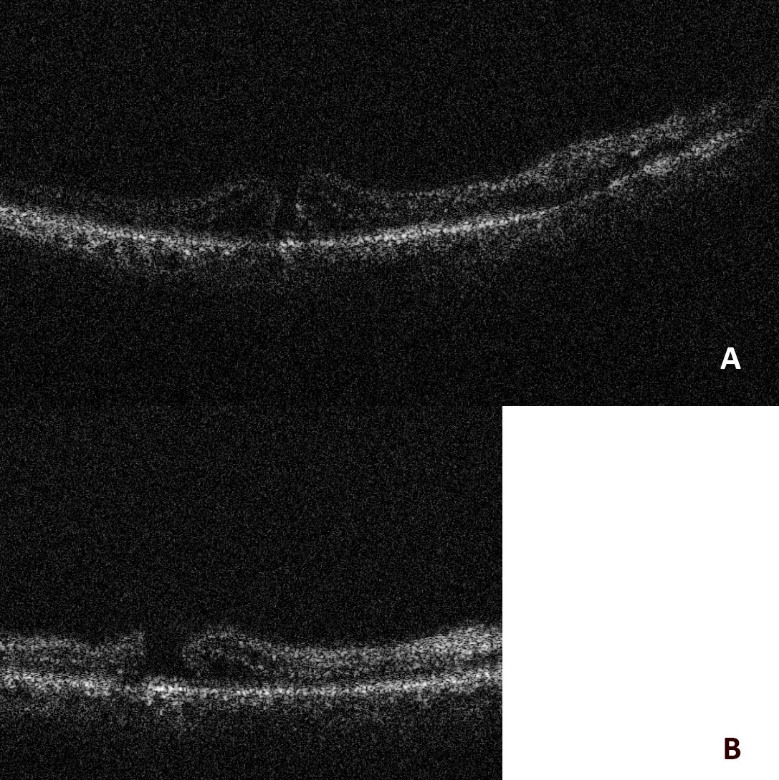
**(A)** Prior to ILM peeling; **(B)** Post ILM peeling - the paramacular thickness has decreased

Lastly, we could confirm a complete ILM peel in a case of macular hole. The IOCT showed a decrease in paramacular thickness, as shown in **Fig. 3**. 

## Discussion

The incorporation of OCT in the operating room is expected to yield positive results due to its high resolution and quick scans. Earlier studies have reported the usefulness of IOCT in cases like vitreomacular traction, macular holes and retinal detachment [**6**-**9**]. The PIONEER (Prospective Intraoperative and Perioperative Ophthalmic Imaging with Optical Coherence Tomography) study in 2014 was the first large scale study to demonstrate the benefit of IOCT in an ophthalmic surgical setting [**4**]. Subsequently, the DISCOVER (Determination of Feasibility of Intraoperative Spectral Domain Microscope Combined/ Integrated OCT Visualization during EnFace Retinal and Ophthalmic Surgery) study, in 2018, further demonstrated the use of this technology in specific cases [**10**]. 

The IOCT altered the surgeon’s decision in the posterior segment between 29.2% and 43% in the DISOVER and PIONEER studies, respectively [**5**,**10**]. Our study agrees with this assessment, having a rate of 26.2%. This modality was especially useful in young adults with adherent vitreous attachments. Induction of posterior vitreous detachment in such cases can be quite difficult and can be missed. If vitreous strands are cut superficially, a fine layer of vitreous remains adherent to the underlying posterior pole near the disc and the macula. 

Additionally, IOCT helped us in cases of epiretinal membranes (ERMs) and tractional retinal detachments (TRDs), like in previous studies [**5**]. The device offered fine details of retinal tissue and overlying membranes helping identify a complete membrane peel. Similarly, during vitrectomy of diabetic TRDs, the system helped clear delineation of tissue planes for efficient and accurate dissection of tissues. Lastly, the IOCT helped confirm a complete reattachment of the retina at the end of surgery.

Other cases of use for this novel system have been demonstrated, including anterior segment surgeries like keratoplasty, Descemet’s membrane stripping endothelial keratoplasty [**8**], and subretinal injection of stem cells of gene therapy [**11**,**12**]. Future studies are still required to understand the complete use of the IOCT system.

This study had various limitations that need discussing. Firstly, this was a pilot study of a prospective long-term study and therefore, the sample size was limited. Secondly, the modality is still relatively novel and understanding the system can take time. Lastly, the surgeon had full knowledge of IOCT systems availability and therefore it may have biased him towards the use of the system.

## Conclusion

The IOCT is a feasible imaging technology for various kinds of vitreoretinal disorders that can significantly affect surgical plans. 

**Conflict of Interest statement**

Authors state no conflict of interest.

**Informed Consent and Human and Animal Rights statement**

Informed consent has been obtained from all individuals included in this study.

**Authorization for the use of human subjects**

Ethical approval: The research related to human use complies with all the relevant national regulations, institutional policies, is in accordance with the tenets of the Helsinki Declaration, and has been approved by the Institutional Review Board of Layton Rahmatullah Benevolent Trust, Lahore, Pakistan.

**Acknowledgements**

None.

**Sources of Funding**

None.

**Disclosures**

None.
